# Disentangling interoception: insights from focal strokes affecting the perception of external and internal milieus

**DOI:** 10.3389/fpsyg.2015.00503

**Published:** 2015-05-01

**Authors:** Blas Couto, Federico Adolfi, Lucas Sedeño, Alejo Salles, Andrés Canales-Johnson, Pablo Alvarez-Abut, Indira Garcia-Cordero, Marcos Pietto, Tristan Bekinschtein, Mariano Sigman, Facundo Manes, Agustin Ibanez

**Affiliations:** ^1^Laboratory of Experimental Psychology and Neuroscience, Institute of Cognitive Neurology, Favaloro UniversityBuenos Aires, Argentina; ^2^UDP-INECO Foundation Core on Neuroscience, Diego Portales UniversitySantiago, Chile; ^3^National Scientific and Technical Research CouncilBuenos Aires, Argentina; ^4^Physics Department, University of Buenos AiresBuenos Aires, Argentina; ^5^Medical Research Council-Cognition and Brain Sciences UnitCambridge, UK; ^6^Department of Psychology, University of CambridgeCambridge, UK; ^7^Laboratory of Neuroscience, Universidad Torcuato Di TellaBuenos Aires, Argentina; ^8^ARC Centre of Excellence in Cognition and its DisordersSidney, NSW, Australia; ^9^Universidad Autónoma del CaribeBarranquilla, Colombia

**Keywords:** interoception, interoceptive awareness, peripersonal space, lesion, stroke, interoceptive sensitivity, exteroception

## Abstract

Interoception is the moment-to-moment sensing of the physiological condition of the body. The multimodal sources of interoception can be classified into two different streams of afferents: an internal pathway of signals arising from core structures (i.e., heart, blood vessels, and bronchi) and an external pathway of body-mapped sensations (i.e., chemosensation and pain) arising from peripersonal space. This study examines differential processing along these streams within the insular cortex (IC) and their subcortical tracts connecting frontotemporal networks. Two rare patients presenting focal lesions of the IC (insular lesion, IL) or its subcortical tracts (subcortical lesion, SL) were tested. Internally generated interoceptive streams were assessed through a heartbeat detection (HBD) task, while those externally triggered were tapped via taste, smell, and pain recognition tasks. A differential pattern was observed. The IC patient showed impaired internal signal processing while the SL patient exhibited external perception deficits. Such selective deficits remained even when comparing each patient with a group of healthy controls and a group of brain-damaged patients. These outcomes suggest the existence of distinguishable interoceptive streams. Results are discussed in relation with neuroanatomical substrates, involving a fronto-insulo-temporal network for interoceptive and cognitive contextual integration.

## Introduction

Interoception is the processing of the body's physiological condition (Craig, 2002), including varied multimodal signals sensed by internal baroreceptors and chemosensors, as well as by surface temperature receptors and nociceptors (Cameron, 2002; Craig, 2002; Garfinkel and Critchley, Craig). The representation of the organism's internal state has been termed interoceptive awareness (Craig, 2009), as it drives goal-directed actions associated with homeostatic regulation (Craig, 2007). Converging neurobiological evidence points to the insular cortex (IC) as a critical hub underlying multimodal interoceptive integration (Saper, 1982; Critchley et al., 2004; Pollatos et al., 2007b; Kurth et al., 2010; Kelly et al., 2012; Farb et al., 2013; Simmons et al., 2013). Topographic and modality-specific signals are relayed by the posterior insula and integrated in the anterior insula, where they interact with information from other limbic and cortical areas (Craig, 2003b)—heartbeat and breathing rate signals being the core of internal information needed for survival.

The IC has been implicated in interoceptive processes, such as awareness of bodily sensations (Khalsa et al., 2009), and exteroceptive processes, such as perception of pain (Brooks et al., 2002; Gramsch et al., 2014), smell (Kurth et al., 2010), and taste (Gagnon et al., 2014; Iannilli et al., 2014; Parabucki and Netser, 2014; van den Bosch et al., 2014). The posterior and mid insular cortices (Kurth et al., 2010) are activated by these processes, especially interoceptive ones. Interoceptive, exteroceptive, and emotional domains overlap in the anterior insula (Kurth et al., 2010), suggesting an underlying commonality (Critchley et al., 2002). In fact, the insula has been proposed as a convergence point between internal and external milieus (Ibanez et al., 2010).

Though generated in the external environment, pain and chemical signals involve a certain degree of body-mapping. This entails cross-modal processing of peripersonal space (Andre et al., 2000; de Paepe et al., 2014; Senkowski et al., 2014), i.e., the immediate surroundings of our bodies (Rizzolatti et al., 1997), which are represented differently from extrapersonal space (Holmes and Spence, 2004). The capacity to encode and integrate information from peripersonal space is vital to behavior and social interactions (Kennedy et al., 2009; Herz, 2014), such as avoidance movements (Berlucchi and Aglioti, 1997; Graziano, 1999; Graziano et al., 2000) and complex behaviors contributing to survival (Greenspan et al., 1999; Verhagen et al., 2004; Farrell et al., 2006).

The anterior insula supports more abstract encoding of internal–external information which interacts with other processes, such as emotion (Paulus et al., 2003; Simmons et al., 2004, 2006). Thus, this structure may support the integration of interoceptive and exteroceptive signals and their contribution to emotional processing networks (Simmons et al., 2013). Such integrative mechanism may rely on smell, taste, and pain, all of which contribute to socio-emotional processes (Critchley and Harrison, 2013; Craig, 2014; van Stralen et al., 2014). This is well-supported by reports of insular activation during emotion and risk-related processing (Simmons et al., 2006) and by evidence highlighting the role of exteroceptive and body-mapped signals in the neural representation of the body and peripersonal space (Azanon and Soto-Faraco, 2008; Mazzola et al., 2009; Azanon et al., 2010). In sum, pain, taste, and smell information may be integrated by insular networks in a peripersonal-like fashion and then further processed by emotional awareness and social behavior mechanisms.

Hence, insular networks for body perception could presumably underlie sensing of (a) a core group of interoceptive sensations that are centered on internal viscera and blood composition; and (b) taste, smell, and pain sensations, which jointly trigger multimodal bodily sensations and interoceptive awareness. This study aims to test a model of multiple interoceptive signaling streams by disentangling the internal and external pathways of body awareness. We evaluated two patients, one with a focal lesion to the right insular cortex (IC), and another with a lesion to the right posterior putamen (including subcortical white matter connecting the posterior IC to the fronto-temporal nodes). The patients' performance in these perception domains was compared with that of healthy controls and other groups of brain-damaged patients.

### External perception

Following Sherrington's pioneering definition (1900), exteroception includes vision, audition, smell, taste, and touch. Interoception might involve signals related to at least three of these senses: smell, taste, and pain. Different IC regions were revealed as primary or secondary areas where these signals are initially processed and passed on for integration (Mufson and Mesulam, 1982; De Araujo et al., 2003). Since the IC constitutes a crucial hub for interoception (Verhagen, 2007; Craig, 2009), internal (visceral) and external (bodily) signals may be sub-served by hubs of the interoceptive network. In this regard, affective and motivational aspects inherent to thermal pain, taste, and olfaction (Greenspan et al., 1999; Wicker et al., 2003; Verhagen et al., 2004; Verhagen, 2007) differ from classical exteroceptive (e.g., visual, auditory) stimuli. The former depend more closely on bodily needs and correspond to primary evolutionary requirements. Accordingly, they are associated with emotional processes and their neural substrates have developed earlier in evolutionary time (Mesulam, 2000). In line with recent approaches that relate body feelings and visceral perception with embodied cognition (Herbert and Pollatos, 2012; Tajadura-Jimenez and Tsakiris, 2014), we propose that the external signals might also be considered as body-mapped signals of an interoceptive peripersonal space. In other words, taste, smell, and pain signals could be conceived as an extension of interoceptive processing to peripersonal space (Ferri et al., 2013).

In functional neuroanatomical terms, taste, smell, and pain sensations engage paralimbic (and mesocortical, including IC) areas and are transmitted through parallel pathways to cortical sites (Verhagen, 2007) involved in autonomic, emotional, and drive functions. This is supported by the functional topography of the IC (Mesulam and Mufson, 1982a,b; Mesulam, 2000) and its segregation into different functional and anatomical connectivity clusters (Kurth et al., 2010; Kelly et al., 2012). Moreover, chemosensation evolved alongside the hypothalamic structures that sense the internal milieu components pertinent to homeostasis (Mesulam, 2000). Taste and smell impairments have been observed in left IC lesions (Pritchard et al., 1999; Cereda et al., 2002), and taste stimuli were reported to activate the IC (Faurion et al., 1999; for a review see, Small et al., 1999). Additionally, heat pain sensation has been proposed as a body signal that motivates emotional behavior and contributes to monitoring the body's physiological condition (Craig, 2002, 2014; Singer et al., 2009). Evidence for this function comes from IC lesion studies reporting pain symptoms (Cereda et al., 2002) and functional connectivity studies showing differential links between the IC and the affective/discriminative pain systems (Peltz et al., 2011). Such functional evidence indicates that taste, smell, and pain are closely related with the internal body signals fostered by IC networks. However, it remains unclear which qualities of taste and smell are simultaneously affected following an IC lesion. To date, no report has assessed these qualities in combination with heat pain thresholds in an evaluation of the body-related external signals.

### Internal interoception

A reliable measure of internal drive is cardiac interoception, which relies on different pathways conveyed to the insular, secondary somatosensory (S2), and anterior cingulate cortices (ACC). The self-heartbeat detection (HBD) is a valid method to quantitatively measure cardiac interoception (Craig, 2003a; Critchley et al., 2004). Functional evidence from electrophysiological studies (Pollatos et al., 2005), intracortical recordings in monkeys (Caruana et al., 2011), and functional magnetic resonance imaging (fMRI) in humans (Dosenbach et al., 2007; Seeley et al., 2007; Seeley, 2008; Sridharan et al., 2008; Taylor et al., 2009; Menon and Uddin, 2010; Deshpande et al., 2011; Kelly et al., 2012) has revealed the involvement of the IC in heartbeat sensitivity. Most studies have successfully used HBD tasks as behavioral measures of cardiac interoceptive sensitivity (Schandry, 1981). Thus, HBD assessment triggers the internally driven interoceptive signals. Interoception has been proposed to encompassed multiple dimensions (Garfinkel and Critchley, 2013) including: (i) interoceptive sensitivity (IS)—the objective detection of visceral sensations, via tasks such as HBD—, and (ii) metacognitive interoception (MI)—reflexive beliefs and thoughts about one's own body sensations. MI and IS represent different interoceptive processes (Garfinkel and Critchley, 2013) they are not necessarily associated (Antony et al., 1995; Zoellner and Craske, 1999) and it is the former the one to which we refer with the present results.

### Disentangling the internal and external sources of interoception

Given their similarities in functionality and gross neuroanatomical location within the IC, internal and external body perception can be functionally related. Here we aim to disentangle external (taste, smell, and pain) and internal (cardiac) body perception signals arriving to the IC by evaluating two rare patients with focal lesions of the (a) right IC and (b) right posterior IC connections to the fronto-temporal nodes. These patients—already evaluated by Couto et al. (2013c) regarding social cognition—were assessed for smell, taste, thermal-pain sensation, and cardiac interoception. Note that lesion studies, as a tool for inferring brain function, are powered by the use of a second control group including patients with damage to areas not implicated in the function of interest (Rorden and Karnath, 2004). Hence, we compared our IL and SL patients with both healthy subjects and non-insular brain-damaged patients. Note that the definition of interoception presently adopted is based on the one posited by Craig (2002). Current neurophysiological and neuroscientific research has not yet enabled a definite consensus on the classification and precise borders of this concept. Indeed, interoception remains as one of the open fields at the frontiers of the neuroscience.

## Materials and methods

### Participants

#### Insular lesion (IL) patient

**Figure 1:**
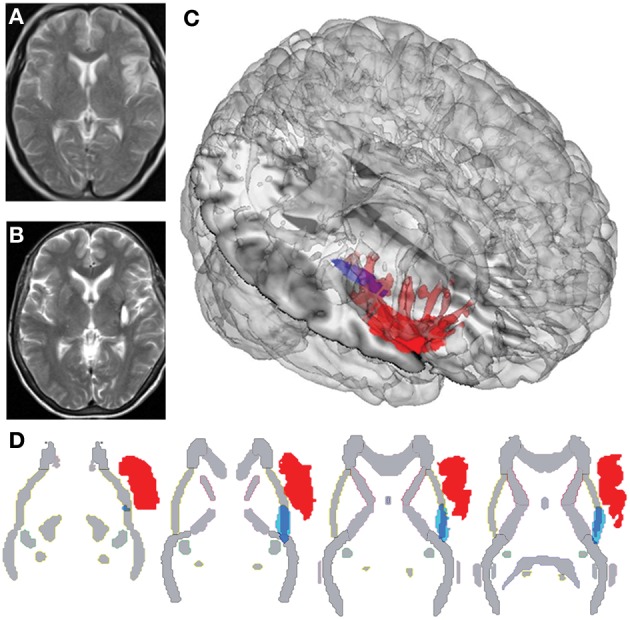
**Different plots of IL and SL brain damage localization**. **(A)** Structural MRI of IL, with sequence T2 showing the right insular cortex lesion. **(B)** Structural MRI of SL, with sequence T2 showing damage to the right posterior putamen, capsula extrema, *claustrum*. **(C)** Three-dimensional rendering of lesion-traced MNI-normalized brain lesions of IL and SL plotted onto a standard T1 glass brain with the Mango software. **(D)** Overlap of lesion-traced MNI-normalized brain lesions of IL and SL and the JHU-Atlas of white matter, showing differential affectation of external capsule in both lesions.

G.G. is a 51-year-old right-handed woman who suffered an ischemic IC stroke 18 months before the evaluation. Her initial symptoms were dysarthria, left hand hemiparesia, and left hemianesthesia. This symptomatology was transient and disappeared 3 days after the onset of the stroke, with no residual signs at neurological examination, despite complaints of a subjective change in taste perception and occasional mild pain in her left arm. Structural magnetic resonance imaging (MRI) of the brain, scanned between 6 and 12 months after the stroke, showed an ischemic focal lesion comprising the complete right anterior, mid, and posterior IC as well as the internal portion of the posterior part of the frontal opercula (fronto-opercular/insular) (Sridharan et al., 2008; Menon and Uddin, 2010; Cauda et al., 2011), with no impairment of the adjacent subcortical structures, as demonstrated by Couto et al. (2013c) (Figures 1A,C,D). Both patients completed general neuropsychological tests (measures of cognitive screening, ACE-R; executive functions, IFS; and intelligence, WAT), as previously reported (see Table 1 and Supplementary material in Couto et al., 2013c).

Table 1**Demographic and neuropsychological assessment**.

**Table:** 

**(A) HEALTHY CONTROL GROUP**																	
	**IL**						**SL**						**Healthy controls**	
		***t***		***p***		***Z-cc***		***t***		***p***		***Z-cc***	
**SOCIODEMOGRAPHIC DATA**													
Age	51	−1.21		0.14		−1.291	59	0.02		0.49		0.02	*M* < 58.86; *SD* < 6.09 (51–70)
Formal education ^#^	17	0.32		0.38		0.347	7^*^	−3.45		0.01^*^		−3.69	*M* < 16.14; *SD* < 2.48 (12–18)
	***t***		***P***	***Z-cc***	***P***	*** Z-ccc***		***t***	***p***	***Z-cc***	***P***	*** Z-ccc***	
**IFS**													
Total score	26/30	0.66	0.27	0.70	0.02^*^	3.75	29/30^*^	4.61	<0.01^*^	4.93	0.02^*^	5.24	*M* < 25.50; *SD* < 0.71 (25–27)
**AFFECTIVE SCREENING**													
Depression (BDI)	3	−0.99	0.18	−1.06			24	2.74	0.02^*^	2.93			*M* < 8.57; *SD* < 5.26 (3–19)
Anxiety state (STAI-S)	21	−2.07	0.04^*^	−2.21	0.07	−2.21	28	−0.58	0.29	−0.62	0.36	−0.64	*M* < 30.71; *SD* < 4.39 (26–39)
Anxiety trait (STAI-T)	28	−1.12	0.15	−1.19	0.34	−0.55	55	1.43	0.10	1.52	0.13	−2.27	*M* < 39.86; *SD* < 9.94 (27–59)

**Table:** 

**(B) STROKE CONTROL GROUP**													
	**IL**						**SL**						**Frontal Damage**	
		***t***		***p***		***Z-cc***		***t***		***p***		***Z-cc***	
**SOCIODEMOGRAPHIC DATA**													
Age	51	−0.16		0.44		−0.17	59	0.16		0.44		0.17	*M* < 55; *SD* < 23.05 (23–78)
Formal Education ^#^	17	1.00		0.19		1.09	7	−1.49		0.10		−1.64	*M* < 13; *SD* < 3.67 (7–16)
**IFS**													
	***t***		***P***	***Z-cc***	***P***	*** Z-ccc***		***t***	***p***	***Z-cc***	***P***	*** Z-ccc***	
Total score	26/30	0.96	0.20	1.05	0.13	2.06	29/30	1.76	0.08	1.93	0.17	1.59	*M* < 22.40; *SD* < 3.42 (19–27)
**AFFECTIVE SCREENING**													
Depression (BDI)	3	−1.16	0.16	−1.27			24	1.00	0.19	1.10			*M* < 14.25; *SD* < 8.87 (5–25)
Anxiety state (STAI-S)	21	−1.86	0.07	−2.04	0.13	−2.05	28	−0.59	0.29	−0.65	0.34	−0.64	*M* < 31.25; *SD* < 5.02 (25–39)
Anxiety trait (STAI-T)	28	−1.17	0.15	−1.28	0.43	−0.27	55	1.76	0.08	1.93	0.09	2.52	*M* < 38.75; *SD* < 8.41 (31–52)

*M, mean; SD, standard deviation, range in parentheses*.

# *In years*.

* *Significantly different to controls*.

#### Subcortical lesion (SL) patient

N.F. is a 59-year-old, right-handed woman who presented with a stroke that had occurred 12 months before the evaluation. Her initial symptoms consisted of left-sided hemiparesia and hemianesthesia, both of which remained for 4 months and then disappeared. At the time of evaluation, she presented with no neurological deficits and complained only about some pain in her left arm, leg, and foot. Brain MRIs, scanned between 6 and 12 months after the stroke, showed a right subcortical hemorrhage. Once normalized to an MNI (Montreal Neurology Institute) standardized brain atlas, the lesion demonstrated engagement of the right putamen and claustrum and the white matter belonging to the external capsule. An additional overlap with the JHU-Atlas of white matter showed damage to the external capsule (Couto et al., 2013c) (Figures 1B,C,D).

#### Control samples

Seven right-handed women with no history of neurological or psychiatric conditions were evaluated as controls (Table 1A). A second control group consisted of five patients presenting brain lesions in the frontal lobe and postcentral gyrus (see Figure S1 and Table 1B). Their demographic data were statistically controlled (see the socio-demographic and neuropsychological results below) in both controls groups.

All the participants signed an informed consent before the evaluation. The study was conducted in accordance with the Declaration of Helsinki and was approved by the institutional ethics committee.

### Assessment

The neuropsychological and clinical evaluations of the patients and healthy controls (including assessment of executive functions, depression, and anxiety) have been described by Couto et al. (2013c). They are briefly recapped in the Results and Supplementary Data sections. The patients' assessments included tasks and measures of olfaction, taste, thermal pain, and cardiac interoception (see below). The subjects were asked to refrain from smoking, eating or drinking anything other than water for 1 h prior to testing.

#### External signals of interoception

##### Smell testing

To establish odor sensitivity thresholds, we used eight solutions at increasing concentrations of phenyl ethyl alcohol in a staircase procedure based on the design of the commercial Sniffin' Sticks (©2014 US Neurologicals, Poulsbo, Washington, USA; Hummel et al., 1997). Odor identification skills were assessed through the commercial test of olfactory function Brief Smell Identification Test (Doty et al., 1984), consisting of 12 stimuli with a forced-choice answer. Finally, threshold and identification means were used to create a global score variable representing overall smell performance. Single *t*-tests between each patient and each control group were calculated using these variables.

##### Smell threshold

Individual odor sensitivity was assessed by acquiring thresholds for phenyl ethyl alcohol with an ascending double-forced choice staircase procedure. We used an eight-step geometric series, starting from a 4% phenyl ethyl alcohol solution (dilution ratio 1:2 in deionized water). Each subject was presented for 3 s at a distance of 3 mm from each nostril with two bottles in a randomized order: one contained only the deionized water, and the other contained the odorant at a certain dilution. While blindfolded, the subjects were asked to identify the odor-containing bottle. The threshold was defined as the trial in which the participant correctly identified five consecutive stimuli (Hummel et al., 1997) and this number was later transformed to percentage of intensity of perceived smell.

##### Smell identification

Odor identification abilities were further evaluated through the B-SIT (B-SIT, Sensonics Inc.). This test consisted of 12 stimuli, each presented for 3 s at 3 mm from each nostril. Each participant selected which odor was perceived from a forced-choice list with four options. The smell identification score was measured as the number of correct choices, ranging from 0 to 12, with higher scores indicating better identification. The 12 odors commonly used in commercially available tests were smoke, chocolate, onion, strawberry, gasoline, turpentine, banana, pineapple, cinnamon, soap, lemon, and rose. The number of correct responses was later transformed into an identification percentage.

##### Taste testing

###### Taste intensity

To evaluate taste intensity perception, each participant was given five sapid stimuli at four increasing concentrations: sucrose (0.03, 0.1, 0.3, 1.0 M), sodium chloride (NaCl; 0.03, 0.1, 0.3, 1.0 M), citric acid (0.001, 0.003, 0.01, 0.032 M), quinine hydrochloride (QHC1; 0.00003, 0.0001, 0.0003, 0.001 M), and monosodium glutamate (Glut; 0.006, 0.02, 0.06, 1.8 M). Each stimulus was dissolved in distilled water and presented at room temperature as part of an ascending concentration series (Bartoshuk et al., 1985). With the subject's tongue extended and stabilized between the lips, each stimulus was applied to both sides of the anterior tongue using a sterile, cotton-tipped applicator. Participants used a number line (range < 0–10) to report the intensity of the stimulus before retracting their tongue. Subjects were told that the first stimulus of each concentration series, distilled water, rated zero on the taste intensity scale. The output score was intensity feeling (from 1 to 50), which was later transformed to percentage of intensity of perceived taste.

##### Taste identification

To measure the subjects' ability to identify five basic tastants, the maximum concentrated stimuli from the previous task (0.3 M sucrose, 0.3 M NaCl, 0.01 M citric acid, 0.0003 M QHC1, and 1.8 M Glut) or distilled water was applied to the tongue using the same procedure described above. Each side of the tongue was tested two times for the five tastants. Participants indicated the perceived flavor by pointing to a labeled card in a six-option forced choice: salty, sweet, sour, bitter, umami, or non-flavor. This test was conducted twice for each stimulus following procedures described elsewhere (Pritchard et al., 1999). The output score was correct responses from 1 to 10, which was transformed into percentage of smell identification.

Finally, taste intensity and identification measures were used to create a global score variable representing overall taste performance. Single *t*-tests between each patient and each control group were calculated using these variables.

##### Thermal testing

Using a Peltier-driven thermo test device (probe size 3 × 3 cm; TSA-II NeuroSensory Analyzer, Medoc Advanced Medical Systems, Rimat Yishai, Israel), we assessed the subjects' threshold for detecting innocuous warmth and innocuous cold, as well as pain thresholds for noxious heat and noxious cold. The Peltier probe was fixed with a rubber band over the skin of the thenar region of each palm and the dorsomedial region of each foot. Temperature stimuli were applied with a slope of 1°C/s, following the method of limits previously described (Yarnitsky and Sprecher, 1994), in which the temperature detection thresholds and pain thresholds were determined as the average of four and three successive stimuli, respectively. The participant stopped these stimuli by pressing a button, with automatic safety limit temperatures of 0°C for the cool/cold and 50°C for the warmth/heat tasks, respectively. The resulting mean stimulation temperatures of the distinct conditions were 37.06 ± 1.52°C for innocuous warm, 23.96 ± 2.60°C for innocuous cold, 43.58 ± 1.93°C for noxious heat, and 11.60 ± 3.09°C for noxious cold. Finally, general scores were calculated for cool sensation, warm sensation, heat pain, and cold pain by averaging outputs of the four limbs. Moreover, we created three global score variables representing: (i) whole pain (the average of heat and cold scores); (ii) thermal sensation (the average of warm and cool scores); and (iii) general thermal-pain sensation score, (the average of the previous two). Single *t*-tests between each patient and each control group were calculated using these variables.

#### Internal stream of interoception

##### Interoceptive measures

###### Heartbeat detection task

Two different HBD tasks have been used in the literature: (i) mental tracking paradigms, currently questioned because the working memory load of the task might affect cardiac perception; (Richards and Lorraine, 1996) and (ii) discrimination tasks, where an interference generated by attending simultaneously to cardiac sensation and external stimuli would constitute a confounding factor. We conducted a behavioral HBD task (Couto et al., 2013b; Melloni et al., 2013; Sedeno et al., 2014), in which the participants tracked their own heartbeats by pressing a key under different conditions. Compared with classical HBD tasks, this measure is more sensitive than traditional interoceptive paradigms given that it provides (i) a one-to-one EKG and motor response fitting and (ii) correct and incorrect response measures. Here we report the two most relevant measures. First, as a motor control condition, each patient was instructed to follow an audio recording of a sampled heartbeat. Next, in another block, they were asked to follow their own heartbeat with no external stimulation or feedback (interoceptive condition). To track the synchronization of the responses with the actual heartbeat, the EKG signal was recorded with an *ad-hoc* circuit composed of an AD620 amplifier and a band-pass filter (low 0.05 Hz, high 40 Hz) and then fed as an analog signal to a laptop computer's audio-card. Three Ag/Ag-Cl adhesive electrodes were placed on every participant in lead II positions, together with headphones for audio stimulus delivery. The signal was processed online with a PsychToolbox script, running on the Matlab platform (MathWorks). The two conditions offered (1) a control measure of audio-motoric performance (first condition) and (2) a cardiac interoceptive measure (second condition). Full instructions and data for the HBD task's validation and reliability have been detailed in previously published work from our group (Couto et al., 2013b; Melloni et al., 2013; Sedeno et al., 2014). The results were reported with an interoceptive accuracy index, which is calculated as follows:

(1) $$\frac{\left(\text{Mean }\left|\text{RT}\right|\;-\;n\text{ of Incorrect Taps}\right)}{n\text{ of Total Taps}}$$

The continuous EKG signal was scanned by an *ad-hoc* matlab script which classified correct taps to those which were time-locked to the current heartbeat considering a fixed time window, which depended on the heart-rate of the participant (average -200 and +600 ms); RT (reaction times) were calculated within this time window with respect to the heartbeat, and their absolute values were used; *n* of Taps is the total amount of taps made by the participant during the whole 2-min-long experimental block. This interoceptive score can vary between 0 and 1, with lower scores indicating better interoceptive performance. Heart rate was also calculated, and included as a covariate in the analysis of interoception differences.

The tapping–tracking design used in this study avoids the cognitive overload of complex processes (such as attentional and working memory demands) involved in mental tracking and discrimination paradigms. For instance, the former imposes this burden as subjects must internally count numbers to keep track of heartbeats (Schandry et al., 1986). In the discrimination paradigms (Whitehead et al., 1977; Critchley et al., 2004), participants have to split their attention between their own heartbeats and an external train of stimuli to judge their synchronicity, which results in an interference affecting performance on the HBD task (Richards and Lorraine, 1996). By circumventing these cognitive demands, our methodology offers a more accurate measure of the ability to follow heartbeats sensations. Second, our method records each subject's answers and allows us to separate those synchronized with heartbeats from those not enabling us to calculate the mean reaction time (RT) and use it to calculate the accuracy index that reflects a participants' performance based on the ratio between correct RT and the total amount of heartbeats recorded.

### Procedure

Two expert vascular neurologists (LS and PR) evaluated the patients via a neurological examination. Two other experts in clinical neuroimaging (FM and BC) analyzed the patients' MRI lesion data. Subsequently, the subjects were compared with the both control groups (Tables 1A,B) regarding age, gender, affective state (see Couto et al., 2013c), executive functions (through the INECO Frontal Screening battery Torralva et al., 2009), anxiety trait/state (see Spielberger et al., 1970), and mood state (Beck's Depression Scale (Beck et al., 1996). In addition, we used both sub-domains of sensory tests and additional global scores for the analyses. Note that clinical populations evince threshold changes in their response to heat and cold pain (Valmunen et al., 2009; Averbeck et al., 2013; Ahmad et al., 2014) and in their sensitivity to smell and taste (Mattes et al., 1995; Grossmann et al., 2005; Iranzo et al., 2013). On the assumption that heat/cold pain perception and smell/taste identification are substantially different processes, we analyzed them additionally to the global scores for each sensation. In addition, we described cardiac interoceptive performance by analyzing the HBD scores of a motor control condition and an interoceptive condition. These measures constitute the gold standard to describe interoceptive effects (Pollatos et al., 2009; Dunn et al., 2010; Elsenbruch et al., 2010; Kirk et al., 2011; Ferri et al., 2013).

### Data analysis

#### Behavioral data analysis

To compare the patients' performance with that of the control samples, we used a modified one-tailed *t*-test (Crawford and Howell, 1998; Crawford and Garthwaite, 2002, 2012; Crawford et al., 2009, 2011). This methodology allows an assessment of significance by comparing multiple individuals' test scores with norms derived from small samples (~5 control subjects). This modified test is more robust for non-normal distributions. It effectively controls for Type I errors and proves robust in comparison with other methods (Crawford and Garthwaite, 2012). Additionally, it has been used in several neuropsychological studies (Carlesimo et al., 2007; Hulleman and Humphreys, 2007; Garrido et al., 2009; Kennedy et al., 2009; Straube et al., 2010) to compare varied measurements of a single case with those of a control sample. We also performed inferences for significance of single case results using the BTD-Cov software (Crawford et al., 2011), including depression symptoms (BDI score) as a covariate. Furthermore, we used this same procedure to covariate out the heart rate measure from the interoceptive score. Because we are reporting case studies, only values with *p* < 0.05 were considered statistically significant in all comparisons (i.e., trends were not considered as significant differences). The effect sizes obtained using the same methods are reported as point estimates (*Z_ccc_* as effect size for the modified *t*-test with covariate analysis), as suggested by a previous study (Crawford et al., 2010). Therefore, the results are presented for a simple analysis (no covariates) and followed by the effect size and *p*-values for the BTD-Cov.

#### Physiological data analysis

##### Heartbeat detection task

EKG data were analyzed using *ad-hoc* scripts that included the following steps for each condition in each subject: (1) extracting heartbeat peaks (HB) from the EKG signal using the peakfinder function (Yoder, 2009); (2) tracking and assigning each EKG peak to the relevant keyboard tap (KT) of each participant using a time window that was dependent on heart rate (HR < 69, window = 650 ms; HR < 85, window = 700 ms; HR < 99, window = 750 ms); and (3) calculating the HB-KT, RT, and measures of accuracy from the assignments, as described elsewhere (Couto et al., 2013b; Melloni et al., 2013; Sedeno et al., 2014).

## Results

Neither the IL nor the SL patients showed general cognitive impairments including the frontal lobe and executive functions (the SL patient even outperformed the healthy controls; see Tables 1A,B, as well as Supplementary Material). While their age and mood state were similar to those of controls from both groups (except for the IL patient, who scored lower for anxiety), the SL patient showed higher depressive symptoms score (BDI) (Table 1A and Supplementary Material). Hence, we assessed every single experimental measure entering BDI score as a covariate. Also, in our interoceptive score analyses, we introduced heart rate as a covariate (see Behavioral Data Analysis above). Note that the brain damaged control group did not show deficits relative to the healthy control group in overall variables.

### Assessment of the external stimuli

#### Smell testing

**Figure 2:**
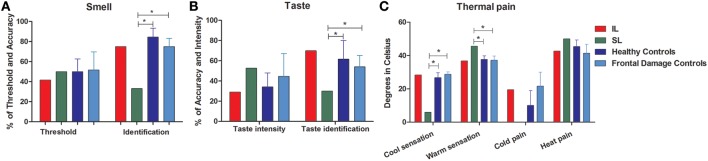
**External stream: smell, taste, and pain**. The figure shows the results of tasks evaluating the external stream of interoception, demonstrating SL patient's impairment and IL patient's normal performance across measures. **(A)** Smell (threshold and identification). **(B)** Taste (intensity and identification). **(C)** Thermal pain (cold perception, warm perception, cold pain, heat pain). ^*^Indicates statistically significant difference.

The IL patient did not differ significantly from controls (Figure 2A) in smell thresholdsor smell (see Figure S2C). The SL patient did not differ significantly from controls in smell thresholds (see Table 2 and Figure S2C) but showed significantly lower smell identification skills (*t* < −5.37, *p* < 0.01, *pcov* < 0.01, *Z_ccc_* < −5.54). Global smell scores were lower for the IL patient than for healthy controls, but such a difference did not stand after covariation (*t* < −2; *p* < 0.05; *pcov* < 0.1; *Z_cc_* < −2.24). The SL patient also showed significantly lower global smell scores, but her impairment remained even after covariation (*t* < −6.21; *p* < 0.01; *pcov* < 0.01; *Z_cc_* < −6.71, see **Table 4**).

**Table 2:** **Results of comparing patient IL and SL with healthy age-matched controls**.

	**Patient IL**						**Patient SL**						**Healthy controls**
	***Score***	***t***	***p***	***p*cov**	***Z-cc***	***Z-ccc***	***Score***	***T***	***p***	***p*cov**	***Z-cc***	***Z-ccc***	
BDI	3	−0.99	0.18		−1.06		24	2.74	0.02^*^		2.93		*M* < 8.57; *SD* < 5.26 (3–19)
Smell threshold	41.6	−0.61	0.28	0.40	−0.65	−0.34	50	0.00	0.50	0.30	0.00	−0.96	*M* < 50; *SD* < 12.73 (42–75)
Smell identification	75	−1.00	0.18	0.20	−1.07	−1.15	33.3	−5.37	<0.01^*^	0.01^*^	−5.75	−5.54	*M* < 84.52; *SD* < 8.91 (67–92)
Taste intensity	29	−0.36	0.37	0.43	−0.39	0.25	52.7	1.25	0.13	0.40	1.35	−0.49	*M* < 34.33; *SD* < 13.68 (20–53)
Taste identification	70	0.42	0.35	0.09	0.45	2.39	30	−1.60	***0.09***	0.01^*^	−1.73	−7.75	*M* < 61.67; *SD* < 18.35 (40–90)
Cool sensation	28.2	0.50	0.32	0.30	0.54	0.71	5.92	−6.70	<0.01^*^	<0.01^*^	−7.16	−7.72	*M* < 26.69; *SD* < 2.90 (21–29)
Warm sensation	36.7	−0.42	0.34	0.36	−0.45	−0.47	45.5	3.55	**0.01**	0.04^*^	3.79	3.86	*M* < 37.66; *SD* < 2.09 (35–41)
Cold pain	19.4	1.00	0.18	0.20	1.07	1.19	0	−1.07	0.16	0.22	−1.14	−1.47	*M* < 10.06; *SD* < 8.79 (1–23)
Heat pain	42.6	−0.64	0.27	0.32	−0.69	−0.63	50	1.12	0.15	0.29	1.19	1.03	*M* < 45.36; *SD* < 3.89 (40–50)
*M*-score	0.05	−0.30	0.39	0.26	−0.33	−1.01	0.03	−0.70	0.26	0.38	−0.77	0.65	*M* < 0.06; *SD* < 0.03 (0.03–0.11)
Interoception	0.5	5.63	<0.01^*^	0.01^*^	6.17	5.71	0.09	−0.61	0.29	0.43	−0.67	−0.34	*M* < 0.13; *SD* < 0.06 (0.05–0.22)

*BOLD font, significant results at p level <0.05; Italic Bold font, trends to significance; BDI, Beck Depression Scale; M, motor condition of heartbeat detection task*.

* *Indicates statistically significant difference*.

When compared with frontal stroke patients, the IL patient showed no differences in either smell threshold or identification. Relative to the same group, the SL patient showed no difference in smell threshold. However, she did evidence impaired smell identification (*t* < −4.57; *p* < 0.01; *pcov* < 0.02; *Z_cc_* < −5, see Table 3 and Figure 2A). In terms of global smell scores, the IL patient showed no significant differences while the SL patient showed significant impairment both with and without covariation (*t* < −2.08; *p* < 0.05; *pcov* < 0.05; *Z_cc_* < −2.28, see **Table 5**).

**Table 3:** **Results of comparing patient IL and SL with frontal damage patients**.

	**Patient IL**						**Patient SL**						**Frontal damage controls**
	***Score***	***T***	***p***	***p*cov**	***Z-cc***	***Z-ccc***	***Score***	***T***	***p***	***p*cov**	***Z-cc***	***Z-ccc***	
BDI	3	−1.16	0.16		−1.27		24	1.00	0.19		1.10		*M* < 14.25; *SD* < 8.87 (5–25)
Smell threshold	41.67	−0.51	0.32	0.46	−0.55	−0.14	50	−0.08	0.47	0.38	−0.09	−0.48	*M* < 51.67; *SD* < 18.07 (42–83)
Smell identification	75	0.00	0.50	0.41	0.00	0.36	33.3	−4.57	0.01^*^	0.02^*^	−5.00	−5.52	*M* < 75; *SD* < 8.33 (67–83)
Taste intensity	29	−0.63	0.28	0.27	−0.69	−0.99	52.7	0.33	0.38	0.35	0.36	0.61	*M* < 44.60; *SD* < 22.61 (10–63)
Taste identification	70	1.28	0.13	0.21	1.40	1.37	30	−1.92	0.06	0.06	−2.11	−2.08	*M* < 54; *SD* < 11.40 (40–70)
Cool sensation	28.25	−0.32	0.38	0.33	−0.36	−0.73	5.92	−15.10	<0.01^*^	<0.01^*^	−16.5	−16.8	*M* < 28.74; *SD* < 1.38 (27-30)
Warm sensation	36.72	−0.17	0.44	0.35	−0.18	0.61	45.5	3.08	0.02^*^	**0.05**	3.38	3.31	*M* < 37.17; *SD* < 2.49 (36-42)
Cold pain	19.43	−0.24	0.41	0.28	−0.26	−0.98	0	−2.36	0.04^*^	0.10	−2.59	−2.35	*M* < 21.57; *SD* < 8.34 (11–29)
Heat pain	42.68	0.22	0.42	0.18	0.24	1.55	50	1.489	0.11	0.23	1.63	1.20	*M* < 41.39; *SD* < 5.28 (34–47)
*M*-score	0.054	−0.27	0.40	0.41	−0.29	0.37	0.03	−0.65	0.28	0.19	−0.71	−1.45	*M* < 0.066; *SD* < 0.041 (0.03–0.13)
Interoception	0.50	4.80	<0.01^*^	0.02^*^	5.26	5.23	0.09	−2.68	0.03^*^	***0.07***	−2.94	−2.72	*M* < 0.237; *SD* < 0.05 (0.19–0.32)
Heart rate	65	−1.86	***0.07***		−1.98		92	1.45	0.11		1.59		*M* < 80; *SD* < 7.54 (67.5–83)

BOLD font and

* *, significant results at p level <0.05;BDI, Beck Depression Scale; M, motor condition of heartbeat detection task*.

*Bold italics indicate trends*.

In sum, as hypothesized at the outset, the IL patient showed no smell impairments relative to frontal damage patients, but she exhibited lower smell performance than healthy controls (this difference, however, disappeared after covariation). Instead, the SL patient was outperformed in smell tasks by both groups. This was the case before and after covariation, a result that also supports our hypothesis.

#### Taste testing

Relative to healthy controls, the IL patient (Figure 2B) showed no impairments in taste sensitivity or recognition (see also Figure S2C). Compared with the same group, the SL patient showed no differences for intensity and a lower taste identification after covariation (*t* < −1.60; *p* < 0.09; *pcov* < 0.01; *Zcc* < −1.73). In terms of global taste scores, the IL patient showed no significant impairments. Neither did the SL patient show any significant deficits (see Table 4).

**Table 4:** **Results of comparing patient IL and SL with healthy age-matched controls**.

	**Patient IL**						**Patient SL**						**Healthy controls**
	***Score***	***t***	***p***	***p*cov**	***Z-cc***	***Z-ccc***	***Score***	***t***	***p***	***p*cov**	***Z-cc***	***Z-ccc***	
Smell	58.33	−2.07	0.05^*^	0.10	−2.24	−2.01	41.67	−6.21	<0.01^*^	<0.01^*^	−6.71	−9.77	*M* < 66.67; *SD* < 3.73 (63–71)
Taste	49.5	0.08	0.47	0.19	0.09	1.38	41.38	−.37	0.37	**0.06**	−0.40	−4.08	*M* < 48.08; *SD* < 16.74 (34–71)
Thermal sensation	32.49	0.44	0.34	0.29	0.47	0.80	25.75	−5.68	<0.01^*^	<0.01^*^	−6.14	−7.19	*M* < 32.01; *SD* < 1.02 (30–33)
Pain	31.05	1.01	0.18	0.19	1.09	1.31	25	−1.02	0.18	0.21	−1.11	−1.64	*M* < 28.04; *SD* < 2.75 (25–32)
Thermal pain sensation	31.77	0.89	0.21	0.20	0.97	1.23	25.38	−2.38	0.03^*^	***0.07***	−2.57	−3.25	*M* < 30.03; *SD* < 1.81 (28–32)
Interoception	0.5	5.63	<0.01^*^	0.01^*^	6.17	6.59	0.09	−.61	0.30	0.43	−0.67	1.33	*M* < 0.13; *SD* < 0.06 (0.05–0.22)

*BOLD font, significant results at p level <0.05; Italic Bold font, trends to significance; BDI, Beck Depression Scale*.

* *Indicates statistically significant difference*.

When compared with the brain damaged group (Figure 2B), the IL patient did not present any impairment in taste intensity or taste recognition. Meanwhile, the SL patient showed impairment neither in taste intensity, nor in taste identification. Global taste scores revealed no significant differences in either the IL patient or the SL patient (see Table 5).

**Table 5:** **Results of comparing patient IL and SL with frontal damage patients**.

	**Patient IL**						**Patient SL**						**Brain lesion controls**
	***Score***	***t***	***p***	***p*cov**	***Z-cc***	***Z-ccc***	***Score***	***t***	***p***	***p*cov**	***Z-cc***	***Z-ccc***	
Smell	58.33	−0.48	0.33	0.48	−0.53	0.08	41.67	−2.08	0.05^*^	0.05^*^	−2.28	−3.27	*M* < 63.33; *SD* < 9.50 (54–79)
Taste	49.5	0.02	0.49	0.43	0.03	−0.28	41.38	−0.89	0.21	0.32	−0.98	−0.72	*M* < 49.30; *SD* < 8.12 (40–58)
Thermal sensation	32.49	−0.63	0.28	0.43	−0.69	0.26	25.75	−9.76	<0.01^*^	<0.01^*^	−10.69	−16.58	*M* < 32.96; *SD* < 0.67 (32–34)
Pain	31.05	−0.18	0.43	0.44	−0.20	−0.24	25	−2.77	0.03^*^	***0.06***	−3.03	−3.00	*M* < 31.48; *SD* < 2.14 (29–34)
Thermal pain sensation	31.77	−0.45	0.34	0.45	−0.49	−0.21	25.38	−6.81	<0.01^*^	**<0.01**	−7.46	−7.94	*M* < 32.22; *SD* < 0.92 (31–33)
Interoception	0.50	4.80	<0.01^*^	0.01^*^	5.26	6.90	0.09	−2.68	0.03^*^^#^	0.05^*^^#^	−2.94	−5.07	*M* < 0.237; *SD* < 0.05 (0.19–0.32)

BOLD font and

* , significant results at p level <0.05;Italic Bold font, trends to significance; BDI, Beck Depression Scale; M, motor condition of heartbeat detection task.

# *Note this effects evidence a better performance of SL regarding the lesion group*.

Therefore, our hypothesis is supported by the absence of impairment in the IL patient, although it does not account for spared performance in the SL patient. However, a qualitative analysis of this latter patient's responses indicated that she misidentified sweet as salty (3/4 times) or bitter (1/4 times), salty as sour (2/6 times), and bitter as salty (3/6 times) or sour (2/6 times), showing a disruption in her subjective taste experiences.

#### Thermal sensation and pain testing

There were no differences between the IL patient and healthy controls (Figure 2C) for thermal cool sensation, warm sensation, heat pain or cold pain (see Table 2). Contrarily, the SL patient showed impairments in thermal cool sensation (*t* < −6.70; *p* < 0.01; *pcov* < 0.01; *Zcc* < −7.16) and warmth sensation (*t* < 3.55; *p* < 0.01; *pcov* < 0.04; *Zcc* < 3.79; see Table 2 and Figure 2C) before and after covariation for depression score on BDI. No further differences were observed. In terms of global thermal sensation scores, the IL patient showed no significant differences while the SL patient exhibited significantly lower performance (*t* < −5.6; *p* < 0.01; *pcov* < 0.01; *Z_cc_* < −6.14). Global pain scores revealed no impairments in the IL patient and no impairment in the SL patient. Finally, regarding global thermal-pain sensation score, the IL patient showed no significant differences but the SL patient showed significantly lower performance, a pattern that did not remain significant after covariation (*t* < −2.38; *p* < 0.03; *pcov* < 0.07; *Z_cc_* <−2.57, see Table 4).

The IL patient and the frontal patients obtained similar scores for thermal cool sensation, warmth sensation, heat pain and cold pain (Table 3 and Figure S2C). Conversely, the SL patient showed impairments in thermal cool sensation (*t* < −15.10; *p* < 0.01; *pcov* < 0.01; *Z_cc_* < −16.54) and warmth sensation (*t* < 3.08; *p* < 0.02; *pcov* < 0.05; *Z_cc_* < 3.38) before and after covariation for depression score on BDI scores. Relative to frontal patients, the SL patient showed no significant differences in heat pain and a difference that did not survive covariation for cold pain (*t* < −2.36; *p* < 0.04; *pcov* < 0.10; *Zcc* < −2.59). Global thermal cool performance was unimpaired in the IL patient but significantly compromised in the SL patient (*t* < −9.76; *p* < 0.1; *p*cov *<* 0.1; *Z_cc_* < −10.6). In terms of global pain scores, the IL patient showed no significant differences but the SL patient showed significantly lower performance, which did not survive after covariation (*t* < −2.77; *p* < 0.03; *pcov* < 0.06; *Z_cc_* < −3.03). Finally, global thermal-pain sensation scores showed no significant differences in the IL patient but were significantly affected in the SL patient (*t* < −6.81; *p* < 0.1; *p*cov *<* 0.1; *Z_cc_* < −7.46, see Table 5).

Considering that cool/cold thresholds are higher as the temperature departs from baseline (diminishes from 32 to 0°C), these results represent a diminished sensitivity to all conditions in SL (cool and warmth sensations).

In sum, when compared with both control groups, the IL patient showed no impairments in taste or thermal-pain sensation, which confirms our hypothesis, but a lower smell performance than healthy controls, which did not survive covariation. Conversely, when compared with healthy controls, the SL patient exhibited impaired smell identification, and diminished sensitivity to cool and warm sensations as well as to global thermal-pain sensation. Such impairments remained in the comparison with the brain-damaged controls, also confirming the hypothesis.

### Assessment of internal stream of interoception

#### Heartbeat detection task (HBD)

**Figure 3:**
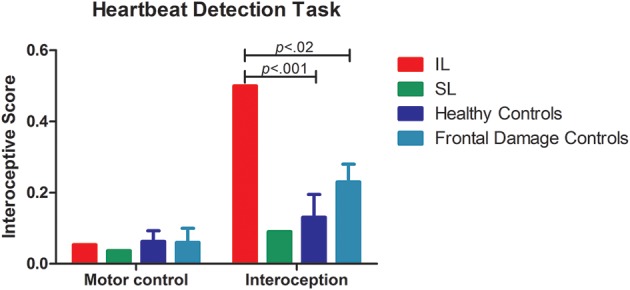
**Internal stream: HBD task**. Results of the accuracy in the HBD task. Left side of the panel shows the motor control condition, where no differences where observed given that all participants performed the task accurately. The right side of the panel shows the performance on interoceptive sensitivity, where the IL patient exhibited impaired interoception. Conversely, the SL patient was spared.

Compared with healthy controls, the IL patient (Figure 3) showed impaired cardiac interoception (*t* < 5.63; *p* < 0.01; *pcov* < 0.01; *Zcc* < 6.17) with preserved performance in the control motor condition (see Table 2). Contrarily, the SL patient's performance was spared in both the interoceptive and the motor control (see Table 2) conditions. Even after covariation for HBD performance, the IL patient had significantly lower scores (*t* < 5.63; *p* < 0.01; *pcov* < 0.01; *Z_cc_* < 6.17) whereas the SL patient performed similarly (see Table 4) to controls.

The patients' differential patterns were replicated following comparison with the brain-damaged group. The IL patient exhibited impairments in the interoceptive (*t* < 4.80; *p* < 0.01; *pcov < 0.02*; *Z_cc_* < 5.26; see Table 3 and Figure 3) but not in the control condition (*t* < −0.27; *p* < 0.40; *pcov* < 0.41; *Z_cc_* < −0.29). Conversely, the SL patient showed preserved performance in both the motor and the interoceptive conditions (she even had better interoceptive performance than the brain-damaged group, but this result did not remain after covariation: *t* < −2.68; *p* < 0.03; *pcov* < 0.07; *Z_cc_* < −2.94; see Table 3 and Figure 3). Similarly, after covariation the IL patient performed significantly worse than brain-damaged patients (*t* < 4.8; *p* < 0.1; *pcov* < 0.1; *Z_cc_* < 5.26). Conversely, relative to brain-damaged patients, the SL patient showed significantly better interoception (see Table 5).

Furthermore, we calculated for both IL and SL patients the heart-rate variability with three different methods and non-significant differences were found compared with healthy controls (see Table S2).

In summary, before and after covariation for HBD performance, and when compared to both healthy controls and the brain-damaged group, the IL patient presented disrupted interoceptive performance, while the SL patient showed no such disruption. Both of these results are in line with the general hypothesis that the internal stream of interoception depends on the insula as its putative basis.

## Discussion

We presented two single cases with respective damage of the right insular cortex (IL) and of right putamen (affecting frontotemporal connections, SL). These patients showed a differential pattern of impairment regarding interoceptive-related behavior and body-mapped functions. The IL patient presented impaired internal (cardiac) interoception and preserved external perception (thermal pain, smell, and taste). A distinct pattern arose in SL, who displayed impaired processing via the external signals (smell identification and thermal-pain thresholds) with preserved cardiac interoception. Importantly, this partially opposite internal–external pattern was replicated when the patients' performance was compared to that of subjects with lesions in other regions. These results suggest that the deficits found in both patients relate to their specific focal lesions, as opposed to unspecific brain damage (Rorden and Karnath, 2004). Second, the pattern of results suggests differential disruption of internal cardiac interoception—affected mainly by focal insular damage (IL)—and external pain-smell—affected by specific subcortical and white matter damage of the fronto-temporo-insular connections (SL). Below we discuss these results in terms of internal and external signals of bodily stimuli and their possible relations with insular networks.

### External stimuli related to interoception

The existence of (external and internal) multimodal insular afferents and their differential requirements for processing (Cameron, 2002; Craig, 2002) supports the view of an external stream (smell, taste, and thermal pain deficits found in SL) involved in interoceptive and IC processing. Chemosensation and pain are typically processed by the paralimbic cortices (ICC, orbitofrontal cortex, ACC, and parahipoccampal cortex) nested between the limbic and higher-order multimodal association regions (Mesulam, 2000; Sewards and Sewards, 2001). Relevant neural pathways run contiguously until they reach the cortical areas, with gustatory pathways ending at the dorsal insula next to the thermal pain region (Verhagen, 2007). Such neurofunctional evidence aligns with the disconnection between IC and frontotemporal regions in the SL patient.

It has been suggested that the information carried by these external stream must first be integrated with stimulus saliency (Seeley et al., 2007; Singer et al., 2009) and hedonic value (Yin and Knowlton, 2006). Thus, disrupted connections in SL might compromise integrative contextual processing of external-internal signals via a fronto-insulo-temporal network including the IC as a critical hub. Damage to this network in the SL patient may underlie ongoing contextual embedding deficits (Mesulam and Mufson, 1982b; Amoruso et al., 2011; Ibáñez and Manes, 2012; Ibanez et al., 2014) leading to impairments in external domains which were spared in the IL patient (see also Couto et al., 2013c). This conjecture might be tested in future studies (Limongi et al., 2014).

### Internal stream of interoceptive afferents

The IL patient exhibited cardiac interoceptive deficits with preserved processing of external signals. Cardiac interoception is a basic modality of visceral perception that relies on an internal drive. It has proven to influence both homeostasis (Oppenheimer et al., 1991, 1992) and affective-cognitive domains (Singer et al., 2009; Garfinkel et al., 2013). Additionally, a wealth of neuroimaging and electrophysiological evidence shows the engagement of the right anterior IC in heartbeat awareness (Craig, 2002; Critchley et al., 2004; Pollatos et al., 2007b; Dunn et al., 2010) and the correlation of this activity with physical and cardio-dynamic variables (Pollatos et al., 2007a). These data point to a critical role of the right IC in sensing cardiac signatures, in line with the cardiac interoceptive impairment evinced by the IL patient. Additionally, the SL patient showed no interoceptive impairment, suggesting that right insula and not their frontotemporal connections running through the external capsule have a specific role in this domain. This is supported by the fact that she outperformed frontal patients, which is to be expected, given that frontal damage affects larger amounts of cortex and white matter than subcortical lesions, and leads to executive deficits (Miller and Cummings, 2007) (see Supplementary Table 1 for comparison between frontal damage and healthy controls, IFS compared with SL: *t* < 1.5; *p* < 0.09; zcc < 1.62).

### Distinct insular networks for processing internal and external streams of bodily signals

As proposed above, cardiovascular and respiratory reflexes (i.e., baroreflex and CO_2_ concentration) that are sensed and processed in a beat-to-beat manner in the brainstem (Barrett et al., 2012) have a highly specific role in physiological modulation, are crucial for motor and affective behaviors (Mesulam and Mufson, 1982b; Garfinkel et al., 2014) and may constitute a privileged internal interoceptive stream. Further evidence suggests they are intrinsically related to the central autonomic regulation of the brainstem, amygdale, and insular cortex (Gray et al., 2009; Feinstein et al., 2013) with scarce signs of engagement from neocortical or higher-order associative structures. Thus, a single and focal right insular lesion might yield interoceptive impairments without compromising the body sensing of external signals.

As expected, the SL patient presented thermal-pain, taste and smell identification deficits. Other lesion studies (Pritchard et al., 1999; Cereda et al., 2002) have shown that IC disconnection from olfactory areas (piriform and mid temporal cortices) is associated with loss of smell. Similarly, cortical thickness of the right insula has been related to odor discrimination, mostly in women (Frasnelli et al., 2010). We also observed spared taste identification in SL that became a significant impairment after covariation with depression symptoms. This can be related with the strong negative correlation (*r* < − 0.85, not reported in results) between depression symptoms and taste functions in the healthy control sample. This impairment was even observed when compared to healthy controls (see Table 4). Thus, our results indicate that two patients presenting selective damage to different areas of the IC body-sensing networks have a differential pattern of disruption of internal and external perception.

### Interoceptive relevance on models of perceptual processing

The possible existence of internal and external subdivisions of interoceptive afferents could reflect a distinction between high and low cognitive processing. The lower level may consist of internal organ signals or proper interoception, such as vegetative cardiac and respiratory rhythms serving vital processes (Oppenheimer et al., 1991). These signals are integrated and represented in the IC (Mesulam and Mufson, 1982b; Brannan et al., 2001; Porges, 2009), shaping cognition in a very direct fashion. For example the activity in IC depends on evoked autonomic response (Critchley et al., 2002). It correlates with performance accuracy in the HBD task (Critchley, 2005) and with changes in peripheral electrodermal activity during a gambling task (Critchley et al., 2000). In addition, the insula is involved in shaping the anticipation and experience of pain and empathetic reproduction of pain experience (Singer et al., 2004). Moreover, the higher level may implicate further connections between the insula and multimodal cognitive association sites (Mesulam, 2000; Couto et al., 2013c) enabling the insula to integrate bottom-up interoceptive signals with top-down predictions from high-order brain regions (i.e., ACC and PFC). This results in the generation of real-time awareness of bodily emotional state (Gu et al., 2013), and contributes to the emergence of complex processes such as moral cognition (Moll et al., 2008), empathy (Decety et al., 2012), or theory of mind (Keysers and Gazzola, 2007).

Conversely, external afferents would involve body-mapped sensory inputs (smell, pain, taste). These may indirectly modulate complex behaviors only after a contextual updating that occurs in the IC just before being projected to cognitive sites (Limongi et al., 2014). This intermediate process may rely on fronto-temporal networks based on their contextual integration to high-level spheres of cognition (Ibáñez and Manes, 2012; Couto et al., 2013a; Baez et al., 2014; Ibanez et al., 2014).

Here we show that the same SL patient who presented emotional awareness deficits (Couto et al., 2013c) is impaired in the external domains of interoceptive processing (chemosensation and thermal-pain). This is consistent with the view that at least some negative emotions, such as disgust, may have emerged from adaptive needs throughout phylogenesis. Note, in this sense, that recent fMRI studies showed insular network activation both when feeling disgust and during observation of another person experiencing this aversive emotion (Wicker et al., 2003). Nevertheless, from the neuroanatomical point of view the affectation of different portions of the insular networks can lead to different patterns of behavioral impairment. Additionally, punctual injury to its white matter connections impact more notably on overall network functionality than damage in one isolated node of the network (Duffau, 2008). This indicates that, within a network, different groups of neurons work together in order to process the same information through designed wad of pathways' connections. Therefore, damage in the subcortical white matter would result in a more consistent affectation of general network functionality relative to the affectation caused by damage to a given gray matter node.

Interoceptive afferent information arriving to the insular cortex, through the lamina I spino-thalamocortical system (lamina 1–solitary tract nucleus–parabrachial nucleus–periaqueductal gray–VMPo thalamus-insula) constitutes the basic information for the elaboration of higher cognitive domains (Craig, 2002) such as verbal memory (Garfinkel et al., 2013), social cognition (Couto et al., 2013b), and emotions (Garfinkel et al., 2014). In particular chemosensation and thermal-pain information are represented by the activity of a fronto-insulo-temporal network and may be anatomo-functionally dissociated through the study of focal lesions in different anatomical points of the network.

### Limitations and further results and future research

This work presents important limitations that should be tackled in future studies.

Interoceptive performance in the brain-damaged group was better than in the IL patient but worse than in the SL patient. These patients' extended damage of the frontal cortex and other regions (reaching adjacent cortical areas and white matter) would explain their intermediate cardiac interoceptive performance. Further studies could assess whether frontal patients present subtle interoceptive deficits and whether these are secondary to other cognitive deficits (e.g., executive dysfunction).

The SL patient showed unimpaired taste abilities, as attested by our methodological strategy. We first used a covariation method to report the variance of the patient's performance beyond the depression covariate. This is a meaningful result in light of the strong negative correlation (*r* < −0.85, not reported in results) between depression symptoms and taste functions in healthy controls. Moreover, results from the four global scores of exteroception evidenced that the patient's impairment is present in thermal-pain processing, especially in heat pain, leaving only taste as a spared domain of external sensation. We then analyzed smell threshold and smell identification separately, and found deficits in the latter. Finally, the SL is not located in the primary gustatory cortex (dorsal anterior IC and dorsal mid-IC, Ogawa et al., 2005; Kurth et al., 2010), which indicates that damage to the taste brain network beyond this critical hub does not compromise the function. In sum, our methodology and results does not enable us to fully rule out a deficit smell domain despite the presence of other exteroceptive impairments.

The IL patient presented spared taste perception, which may seem to contradict the primary role of the IC in gustatory processing (Rolls et al., 2009). Nevertheless, similar findings were observed in the IL patient assessed by Mesulam (2000) and in three out of four focal insular patients evaluated by Cereda et al. (2002). Moreover, gustatory processing relies on a distributed network, with orbitofrontal hubs sub-serving multimodal integration (with visual and olfactory signals) and representation previous to subjective report (Rolls et al., 2003, 2009). In this sense, if gustatory process pertain to the external stream of interoception (as suggested here), it would be more dependent on extended fronto-temporal nodes.

Although we made a covariation by depression scores, we cannot rule out their possible effect on interoception. In particular, smell sensitivity (but not smell identification) is reduced in Major Depressive Disorder (MDD) (Pause et al., 2001; Thomas et al., 2002). Nevertheless, research on mood disorders and olfaction has yielded inconsistent results. In a recent review on olfactory perception and depression (Schablitzky and Pause, 2014), a number of studies showed that reduced performance on smell tasks is disorder-specific (Postolache et al., 1999; Swiecicki et al., 2009; Schablitzky and Pause, 2014). Moreover, negative association (Scinska et al., 2008) and even increased olfactory discrimination during depressive mood states (Goel and Grasso, 2004; Pollatos et al., 2007a) have been reported. Furthermore, studies assessing odor *identification* in MDD patients have shown no differences with healthy controls (Amsterdam et al., 1987; Warner et al., 1990; Kopala et al., 1994; Pause et al., 2003; Lombion-Pouthier et al., 2006; Swiecicki et al., 2009; Negoias et al., 2010; Naudin et al., 2012). However, the performance of our SL patient deviates from previous reports (i.e., reduced olfactory *sensitivity* but preserved *identification* in MDD). In fact, the pattern observed in our SL patient is the exact opposite (compromised *identification* and preserved *sensitivity*). Furthermore, the patient does not present a depression diagnosis, but only some depressive symptoms which were also covariate. Thus, although we cannot rule out the possibility of these symptoms affecting the results in the olfactory tasks, they do not represent the most plausible explanation for the patients' deficit pattern.

The absence of impairments in pain, taste and smell identification in the IC patient could reflect the action of compensatory functions provided by an intact left insula. In fact, this structure has been implicated in pain, taste and smell recognition (Pritchard et al., 1999; Brooks et al., 2002; Cereda et al., 2002). While relatively unexpected, this result also would be explained by the patient's use of explicit compensatory strategies. In addition, enhanced neuroplasticity and a successful functional remapping of the fronto-insular-temporal network after IC stroke would enable correct pain, smell and taste recognition. In fact, this interoceptive information are processed ultimately in the right anterior insula. Such interpretations are in line with our findings in patients with damage in the right network particularly of extra-insular connections.

Finally, the SL is not located in the primary gustatory cortex (dorsal anterior IC), which indicates that damage to the taste brain network beyond this critical hub does not compromise the function. In addition, this interpretation is reinforced by evidence that damage to the left insula causes a bilateral affectation in taste recognition (Pritchard et al., 1999).

Since all perceptive tests require preserved language abilities, our deficits would not be explained by a lack of transmission between the right insular network and neural substrates of language. Indeed, there is evidence that absence of the more important bundle of inter-hemispheric communication does not affect the verbal report of chemosensation awareness (Aglioti et al., 2001).

## Conclusion

Most previous reports of the insular patients (Calder et al., 2000; Adolphs, 2002; Adolphs et al., 2003; Bar-On et al., 2003) included extended neural damage to the amygdala and frontal-parietal-temporal opercula. Focal cerebrovascular accidents represent a gold-standard model for brain injury studies (Rorden and Karnath, 2004). A particular strength of this work is that we compare only patients with very rare focal lesions of the insula and adjacent sites. A differential pattern of behavioral disruption as evidenced by the internal stream's affectation of IC lesion in IL and the external one in SL may shed light on the distinct neuroanatomical signatures of body perception. These disparate deficits would imply a hypothetical stratification of the multimodal bodily signals which surround the body in a peripersonal space, contribute to interoception and engage different aspect of insular networks for coordinating the internal and external milieus with higher functions such as emotional awareness.

### Conflict of interest statement

The authors declare that the research was conducted in the absence of any commercial or financial relationships that could be construed as a potential conflict of interest.
