# Supplementary material for: Disentangling interoception: insights from focal strokes affecting the perception of external and internal milieus
Source: Front Psychol. 2015 May 1;6:503. doi: 10.3389/fpsyg.2015.00503 (PMC4416458; doi:10.3389/fpsyg.2015.00503)
Supplement: Supplementary file 1 [file DataSheet1.DOCX]

**Supplementary Material**

**Methods**

**Heartbeat detection paradigm**

We performed a motor tracking interoception test, the Heartbeat Detection Task (HBD), which has already been validated and applied in a previous study of our group ([Couto, et al., 2013](#_ENREF_1); [Sedeno, et al., 2014](#_ENREF_5)). In the HBD participants are required to tap a computer keyboard along with their heartbeat in different conditions. This motor tracking task was selected based on its differences, and advantages for our research, when compared to traditional interoceptive sensitivity paradigms. In this way, heartbeat discrimination task ([Whitehead, Drescher, & Heiman, 1977](#_ENREF_6)) involve a possible interference factor ([Pennebaker, 1982](#_ENREF_3)) introduced by the nature of the instructions that request participants to attend both to their endogenous heartbeat sensations and to auditory or visual cues. Regarding the other traditional paradigm, mental tracking ([Schandry, 1981](#_ENREF_4)), the HBD has the advantage of measuring correct and mistakes answers.

**Results**

**Sociodemographic, clinical and neuropsychological results**

Sociodemographic, clinical and neuropsychological results are provided in table 1. No significant differences in age (*t* = -1.21; *p* = .14) and years of formal education (*t* = .32; *p* = .38) were present between the IL patient and the control sample. No IL patient-control differences were observed in either the neuropsychological EF evaluation (IFS) (*t* = .66; p = .27) or depression (BDI, *t* = -0.99; p = .38). However, the patient showed a significantly lower score anxiety trait and no differences in anxiety state (STAI-S, *t*= -2.07; p = .04; STAI-T, *t*= -1.12; p = .15).

The age of the SL patient was not different from that of the control sample (*t* =0.02; *p* = .49), but she had a significantly lower formal education level than the controls (*t* = -3.45; *p* =.01). Between the SL patient and control sample, the SL patient was found to have significantly better performance in EF (*t* =4.61; *p* =.01). Higher depression symptoms were found in either SL (BDI, *t* =2.74; *p =*.02) and no differences in anxiety (STAI-S, *t*=-0.58; *p* =.29; STAI-T, *t*=1.43; *p*=.10). Due to the differences in depression symptoms, we performed a covariance test ([Crawford, Garthwaite, & Ryan, 2011](#_ENREF_2)) to analyze the influence of this variable on the performance of specific emotion and social cognition tests. All results for SL are reported after controlling for educational level and are shown as *t* values with one-tailed significances obtained by this method.

**Tables**

**Table.S1.** Comparison between healthy controls and frontal damage controls regarding general neurocognitive and mood symptoms domains.

|  |  | **IL** | | | **SL** | |  | | **Frontal Damage** | | |
| --- | --- | --- | --- | --- | --- | --- | --- | --- | --- | --- | --- |
| **Sociodemographic data** |  | *T* | *p* | *Z-cc* |  | *t* | | *p* | *Z-cc* | |  |
| Age | 51 | -0.16 | .44 | -0.17 | 59 | 0.16 | | .44 | 0.17 | M=55; SD=23.05 (23-78) | |
| Formal Education # | 17 | 1.00 | .19 | 1.09 | 7 | -1.49 | | .10 | -1.64 | M=13; SD=3.67 (7-16) | |

| **IFS** |  | *t* | *P* | *Z-cc* | *P* | *Z-ccc* |  | *T* | *p* | *Z-cc* | *P* | *Z-ccc* |  |  |
| --- | --- | --- | --- | --- | --- | --- | --- | --- | --- | --- | --- | --- | --- | --- |
| Total Score | 26/30 | 0.96 | .20 | 1.05 | .13 | 2.06 | 29/30 | 1.76 | .08 | 1.93 | .17 | 1.59 | M=22.40; SD=3.42 (19-27) |  |
| **Affective screening** |  |  |  |  |  |  |  |  |  |  |  |  |  |  |
| Depression (BDI) | 3 | -0.98 | .20 | -1.10 |  |  | 24 | 0.85 | .23 | 0.95 |  |  | M=14.25; SD=10.24 (5-25) |  |
| Anxiety state (STAI-S) | 21 | -1.58 | .11 | -1.77 | .19 | -1.78 | 28 | -0.5 | .33 | -0.5 | .38 | -0.5 | M=31.25; SD=5.80 (25-39) |  |
| Anxiety trait (STAI-T) | 28 | -0.99 | .20 | -1.11 | .45 | -0.23 | 55 | 1.50 | .12 | 1.67 | 0.14 | 2.18 | M=38.75; SD=9.71 (31-52) |  |
| *M: mean; SD: standard deviation, range in parentheses. # in years. * Significantly different to controls.* | | | | | | | | | | | | | | |

**Table S2.** Results of comparing patient IL and SL hear-rate variability with healthy controls.

|  | | **Patient IL** | | | | | **Patient SL** | | | | | **Healthy Controls** | |  |
| --- | --- | --- | --- | --- | --- | --- | --- | --- | --- | --- | --- | --- | --- | --- |
|  | ***score*** | | ***t*** | ***p*** | ***Z-cc*** | ***score*** | | ***t*** | ***p*** | ***Z-cc*** |  | |  |  |
| **High-frequency** | 42.3 | | -0.07 | 0.47 | -0.08 | 17.7 | | -1.31 | 0.12 | -1.41 | *M= 43.74; SD= 18.07* | | | |
| **Low-frequency** | 57.74 | | 0.13 | 0.45 | 0.14 | 81.42 | | 1.31 | 0.13 | 1.43 | *M= 55.1; SD= 18.33* | | | |
| **Neuro-elf** | 1.48 | | 0.40 | 0.35 | 0.44 | 0.09 | | -0.81 | 0.22 | -0.89 | *M=1.020; SD= 1.027* | | | |

*High-frequency is the Relative power of the specific band (0.15–0.4 Hz) calculated as follows: HF [%] = HF [ms2]/total power [ms2] × 100%.*

*Low-frequency is the Relative power of the specific band (0.04–0.15 Hz) calculated as follows: LF [%] = LF [ms2]/total power [ms2] × 100%*

*Both high and low frequency measures were obtained by processing the R-R interval values of each participant with the Kubios software version 2.0 (Biosignal Analysis and Medical Imaging Group (BSAMIG), Department of Physics, University of Kuopi,oKuopio, FINLAND; http://kubios.uef.fi/KubiosHRV/).*

*Neuro-elf is the measure computed with the MATLAB script "computehrv" from the CC Attribution-Noncommercial-Share Alike web site: http://neuroelf.net/wiki/doku.php?id=computehrv. It represents the power spectral contribution of frequencies in the specified 0.15-0.4Hz range.*

**Figures**

**Fig.S1.** Multislice plot showing the lesions and localization in z axis corresponding to the six patients with brain damage.

**
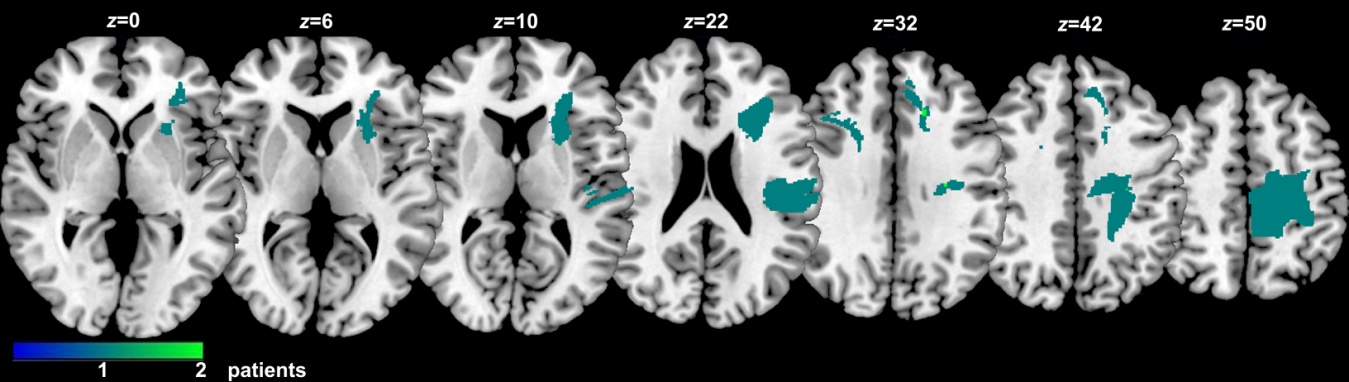
**

**Fig.S2.** Results of tasks evaluating the external stream of interoception showing SL’s and IL’s normal performance. **A.** Smell. **B.** Taste. **C.** Thermal sensation and cold/heat pain.

**
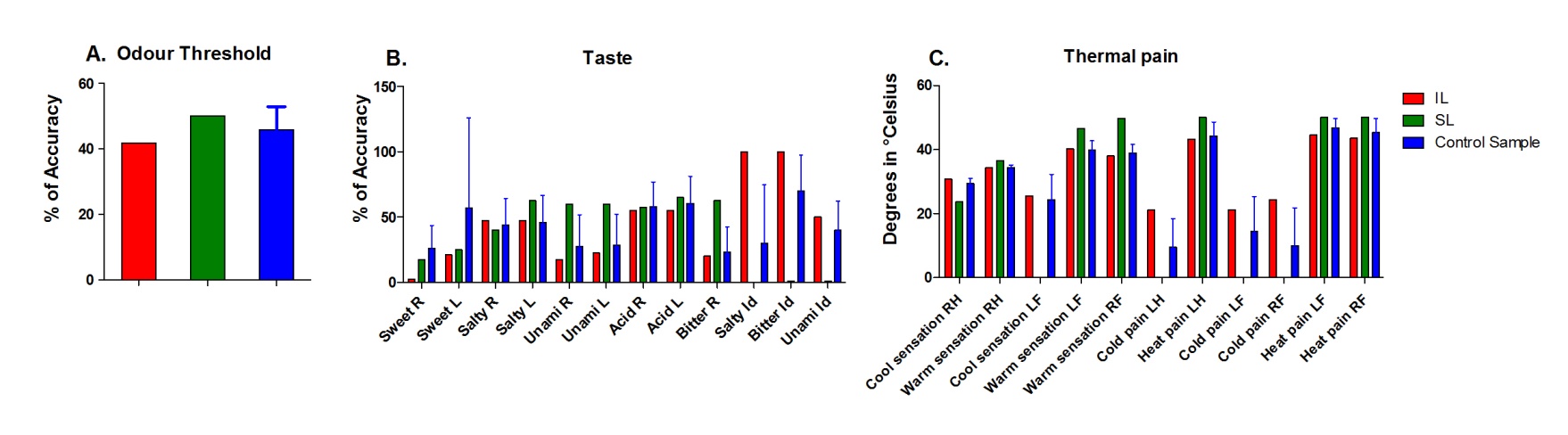
**

References

Couto, B., Salles, A., Sedeno, L., Peradejordi, M., Barttfeld, P., Canales-Johnson, A., Dos Santos, Y. V., Huepe, D., Bekinschtein, T., Sigman, M., Favaloro, R., Manes, F., & Ibanez, A. (2013). The man who feels two hearts: the different pathways of interoception. *Soc Cogn Affect Neurosci*.

Crawford, J. R., Garthwaite, P. H., & Ryan, K. (2011). Comparing a single case to a control sample: testing for neuropsychological deficits and dissociations in the presence of covariates. *Cortex, 47*, 1166-1178.

Pennebaker, J. W. (1982). *The psycholgy of phisical symptoms* New York: Springer.

Schandry, R. (1981). Heart beat perception and emotional experience. *Psychophysiology, 18*, 483-488.

Sedeno, L., Couto, B., Melloni, M., Canales-Johnson, A., Yoris, A., Baez, S., Esteves, S., Velasquez, M., Barttfeld, P., Sigman, M., Kichic, R., Chialvo, D., Manes, F., Bekinschtein, T. A., & Ibanez, A. (2014). How Do You Feel when You Can't Feel Your Body? Interoception, Functional Connectivity and Emotional Processing in Depersonalization-Derealization Disorder. *PLoS One, 9*, e98769.

Whitehead, W., Drescher, V., & Heiman, P. (1977). Relation of heart rate control to heartbeat perception. *Biofeedback Self Regul, 2*, 371 - 392.
